# Analysis of Ancestral Inbreeding Across Main Founders and Ancestors in the Polish Simmental Cattle Population

**DOI:** 10.3390/ani16152404

**Published:** 2026-08-04

**Authors:** Joanna Kania-Gierdziewicz, Agnieszka Otwinowska-Mindur, Olga Jarnecka, Wojciech Jagusiak

**Affiliations:** Department of Genetics, Animal Breeding and Ethology, University of Agriculture in Kraków, al. Mickiewicza 21, 31-120 Krakow, Poland; agnieszka.otwinowska@urk.edu.pl (A.O.-M.); olga.jarnecka@urk.edu.pl (O.J.); wojciech.jagusiak@urk.edu.pl (W.J.)

**Keywords:** inbreeding, founders, ancestors, Simmental cattle

## Abstract

This study estimated the impact of main founders and ancestors on inbreeding subdivision in the Polish Simmental cattle population. Two reference populations were defined: subset A (20,827 cows born 1984–2009) and subset B (19,554 cows born 2010–2020). Ancestral inbreeding coefficients were calculated relative to the main founders and ancestors for all cows in both subsets. Significant changes in ancestor composition were observed in younger cows. The main founders with the highest genetic contribution numbered 10 in subset A and 7 in subset B, with 7 common to both. Main ancestors numbered 11 (all bulls) in subset A and 15 in subset B, with 6 shared between subsets. The frequency of occurrence of common founders in subset B ranged from 42% to 49%, while that of common ancestors ranged from 31% to 68%. Kalinowski’s new inbreeding coefficient was six times higher in subset B than in subset A. Although overall inbreeding levels remained low, the proportion of cows with elevated inbreeding increased over time despite continuous importation of breeding animals. These findings suggest that breeders should scrutinize more carefully the pedigrees of imported animals to prevent further inbreeding accumulation.

## Simple Summary

Inbreeding—the mating of closely related animals—is a growing problem in many livestock breeds, including Simmental cattle raised in Poland for both meat and milk production. When animals share many common ancestors, their offspring more often inherit identical copies of genes, which can reduce health, fertility, and overall productivity. Because importing bulls and cows from abroad was expected to introduce new, unrelated genetics, inbreeding levels in Polish Simmental cattle were assumed to remain low. Our study found, however, that inbreeding has been steadily increasing in younger cows. By tracing the ancestry of the entire Polish Simmental population, we identified the key ancestors responsible for this rise. Our findings show that a small number of highly influential ancestors—many of them imported animals—are closely related to the local population, meaning that importing more animals from the same genetic lines does not reduce inbreeding. We therefore recommend that breeders carefully check the genetic background of imported animals before use, selecting only those that are genuinely unrelated to the existing Polish population. This approach can help preserve the genetic diversity, health, and long-term productivity of Simmental cattle in Poland.

## 1. Introduction

Inbreeding has been increasing in many populations of farm and companion animals. It is defined as the probability that two randomly chosen alleles at the same locus are identical by descent from a common ancestor of the animal’s parents [1]. An effective tool for monitoring changes in genetic diversity at the herd or population level is pedigree analysis [2,3]. Inbreeding and relatedness coefficients have been the most commonly used indicators of genetic variation in a population over many decades. Inbreeding affects the viability of organisms by disturbing their homeostasis and resistance to environmental stress [4]. Numerous studies confirm that heterozygous animals more readily maintain environmental homeostasis, associated with the ease of adapting to environmental conditions [5].

Mate selection can have many positive and negative consequences on the health and survival of future progeny: it leads to the choice of the best individuals as parents of the next generation, and to the elimination of weak and genetically burdened animals, but breeders often select closely related animals for mating, which affects the level of inbreeding in the herd. The use of inbreeding requires strict selection of the progeny. In Japanese Shorthorn cattle, the effective population size decreased from 127.8 in 1980 to 82.6 in 1999 and then stabilized at around 90, highlighting the necessity of maintaining balanced breeding practices to control inbreeding [6].

Rios Utrera et al. [7] estimated the inbreeding coefficients for the Mexican population of Simmental cattle born between 1985 and 2014 and found that those values decreased from 1.65% in 1997 for the pedigree file of 32,357 individuals to 0.668% in 2014 for 54,276 animals. The authors claimed that, despite low inbreeding over the last decade, the genetic variability of that population decreased at the same time, due to pedigree bottlenecks. After analyzing the pedigrees of 27,985 Colombian Simmental cattle born between 1975 and 2017, Amaya et al. [8] found that the average annual inbreeding values decreased from 5.06% in 1980 to 2.25% in 2017. That change, according to those authors, resulted from the introduction of new sires from Europe and North America for breeding which, through the increase in the effective number of founders, contributed to increased genetic diversity. Araujo Neto et al. [9] estimated the inbreeding coefficient (1.49%) and effective population size (48.03) for 77,533 Brazilian Simmental cattle born between 1970 and 2016. They suggested that inbreeding can depress various economically important characteristics of beef cattle, and stressed the importance of monitoring the level of inbreeding when making mating decisions and of importing semen from unrelated bulls. Török et al. [10] reported that after 1997 the annual increase in the inbreeding coefficient of the Hungarian Simmental cattle population was 0.01%, with overall inbreeding of 0.5%. Melka et al. [11] found a 7% decrease in genetic diversity in Brown Swiss cattle over the last four decades (from 1967 to 2007). They found also that the loss of genetic diversity in other Canadian dairy breeds is due to the increasing inbreeding per generation, from 0.8% for Milking Shorthorns to 1.26% for Canadienne breed, and may lead to inbreeding depression [11]. Inbreeding depression occurs when the frequency of homozygous genotypes increases in the population, simultaneously reducing the frequency of favorable heterozygotes [12,13]. At the population level, the consequence of increasing homozygosity is the loss of genetic variability [14]. Accumulation of inbreeding adversely affects many traits important for breeders and leads to the premature culling of animals [15].

Several inbreeding coefficients have been developed to quantify and decompose the degree of inbreeding in a population. The classical inbreeding coefficient (F_C_), introduced by Wright [16] and later extended by Meuwissen and Luo [17] for large pedigrees, measures the probability that two alleles at a given locus in an individual are identical by descent. It is the most widely used measure and forms the basis for estimating inbreeding depression and monitoring population genetic diversity. To allow a more nuanced analysis of the history of inbreeding within a pedigree, Ballou [18] introduced the ancestral inbreeding coefficient (F_Bal_), defined as the probability that any allele in a given animal was identical by descent—that is, autozygous—at least once in previous generations due to inbreeding. F_Bal_ captures the cumulative ancestral exposure to inbreeding regardless of whether those alleles are currently autozygous and is therefore considered an indicator of the potential for purging deleterious recessive alleles. Kalinowski et al. [19] further refined this concept by proposing two complementary coefficients: the ancestral inbreeding coefficient (F_Kal_), which measures the proportion of alleles that are currently identical by descent and that have also been identical by descent in at least one previous ancestral generation; and the new inbreeding coefficient (F_New_), calculated as the difference between F_C_ and F_Kal_. F_New_ therefore represents inbreeding that occurs for the first time in the individual’s pedigree and is expected to be more harmful than ancestral inbreeding, as the deleterious recessive alleles involved have not previously been exposed to purging selection. An additional coefficient, the ancestral history coefficient (AHC), was later proposed by Baumung et al. [20] as a measure of how frequently alleles in an individual’s genome have been in the identical-by-descent state throughout pedigree segregation, providing a broader characterization of the historical inbreeding load. An important conceptual distinction has been proposed: between new inbreeding, where alleles become identical by descent for the first time in an individual, and ancestral inbreeding, where alleles have previously been identical by descent in earlier generations of the pedigree [18,19]. This distinction is relevant because repeated exposure to inbreeding may, over time, contribute to the purging of deleterious recessive alleles through selection against homozygous genotypes [18].

Doekes et al. [13] demonstrated in Dutch Holstein–Friesian cows that new inbreeding was more harmful than ancestral inbreeding for yield traits, providing empirical evidence of purging at the pedigree level: new inbreeding (F_New_) significantly reduced milk, fat, and protein yields, whereas ancestral inbreeding did not. For example, for yield traits a 1%, increase in new inbreeding was associated with a 2.42 kg decrease in 305-day fat yield, compared to a near-zero effect of ancestral inbreeding. Also, Kania-Gierdziewicz et al. [21] found that a 1% increase in the new inbreeding coefficient was associated with a reduction of approximately 70 kg in milk yield, 2.6 kg in fat yield, and 2.3 kg in protein yield per lactation.

The widespread implementation of genomic selection in cattle breeding programs has added new dimensions to the challenge of managing genetic diversity. Doublet et al. [22] reported that while genomic selection substantially increased annual genetic gains in French dairy cattle breeds (by 33–71%, depending on breed), it was associated with a marked increase in the inbreeding rate in international Holstein cattle, reaching 0.55% per year based on runs of homozygosity (ROH). This increase was largely attributed to the intensive use of a small number of elite bulls. Genomic analyses of beef cattle breeds have revealed similarly concerning trends: Kasarda et al. [23] found a significant and continuous linear decline in effective population size across six beef cattle breeds, including Aberdeen Angus, Charolais, Hereford, and Limousin, emphasizing the need for continuous monitoring of genetic diversity to preserve the long-term productive potential of these breeds. Lozada-Soto et al. [24] confirmed in American Angus cattle that although genomic selection generally reduced pedigree inbreeding accumulation rates, significant depressive effects of inbreeding on economically important growth traits were still observed, particularly for recent inbreeding.

There are good ways to control the increase in the level of inbreeding. One is to limit the number of related animals entering the cattle herd. Another is to implement pedigree control of individual animals to avoid high inbreeding rates in the offspring. This means monitoring mate pedigrees and limiting close-kinship mating [25], which helps to avoid the accumulation of lethal recessive alleles in the homozygous state. The best way to prevent a high level of inbreeding in a herd is to apply pedigree analysis combined with appropriate mating plans. The more complete the pedigrees are (containing more information about several generations back), the more reliable are the calculated values of the inbreeding coefficient [26,27]. Pedigree analysis has also been shown to be an effective tool for monitoring changes in genetic diversity in the herd [7,8,27].

Accurate inbreeding estimation depends heavily on the completeness of pedigree records. Sell-Kubiak et al. [28] demonstrated that in the Polish Holstein–Friesian population, comprising over 9.8 million animals, between 30% and 50% of animals born between 1985 and 2015 lacked relevant ancestral information, leading to underestimation of the true inbreeding level. For the year 2015 they estimated that the observed inbreeding level, when accounting for missing parental information, was 3.30%, which was more than twice the value obtained with the classical approach, and they emphasized the need to maintain precise mating records and implement population-wide management strategies to avoid a further rapid growth of inbreeding. In Ecuadorian Charolais cattle, Cartuche-Macas et al. [29] similarly showed that pedigree completeness declined over time and that the strategic use of foreign genetic resources could effectively help mitigate genetic diversity loss and control inbreeding rates, a finding of direct relevance for other populations that rely on imported animals to maintain genetic variability. In the Polish Simmental cattle population, importation of Simmental bulls and cows from other countries is permitted, which should help maintain low inbreeding levels, but Kania-Gierdziewicz et al. [21] found that the number of inbred cows increased over time, indicating that importing Simmental bulls was not effective in reducing inbreeding levels.

In this study we estimated the impact of main founders and ancestors on the subdivision of inbreeding in the Polish Simmental cattle population, to augment previous findings regarding the increase in inbreeding.

## 2. Materials and Methods

The pedigrees of Simmental cows formed the study material. The data came from the Polish national livestock recording system (FedInfo, Warsaw, Poland), obtained from the Polish Federation of Cattle Breeders and Dairy Farmers. Two reference populations (subsets) were created for the analysis. Subset A included 20,827 Simmental cows born between 1984 and 2009, and subset B comprised 19,554 cows born between 2010 and 2020. Simmental cattle in Poland, as a small population, are not subject to genomic selection. We chose the period from 2010 to 2020 due to changes in breeding policy and the increased use of foreign bulls, which was intended to reduce the inbreeding of this small population. The complete pedigree file contained 89,220 animals (cows and their ancestors). The level of pedigree completeness across generations was described in detail by Kania-Gierdziewicz et al. [21]. The potential errors in sire identification within the pedigrees of the Simmental cattle that we studied had been corrected earlier, due to the mandatory molecular verification of parentage for animals qualified for breeding in Poland.

The Simmental cows were daughters of 3033 sires and 30,140 dams. Only 109 cows had no information about their dams. Most sires had between 1 and 10 daughters. The following generation intervals were obtained: 7.43 years for the sire–son path, 6.91 years for the sire–daughter path, 4.99 years for the dam–son path, and 4.81 years for the dam–daughter path. The mean and maximum family sizes for sires were 12.23 and 488 cows, respectively. The corresponding values for dams were 1.34 and 8 cows [21]. Such a long generation interval in father–offspring paths may contribute to an uncontrolled increase in inbreeding and an increase in the number of inbred animals, because a small number of elite bulls (even imported) used for an extended period leave large groups of half-siblings in the population [21].

### Pedigree-Based Analyses

We estimated pedigree completeness according to Boichard et al. [30] as the mean equivalent of complete generations:

$$EqG=\frac{1}{N}\sum_{j=1}^{N}\sum_{i=1}^{n_{i}}\frac{1}{2^{gij}}$$

where

EqG—mean equivalent of complete generations,

N—number of animals included in the analysis,

n_i_—total number of ancestors of j-th animal in the population under study,

g_ij_—number of generations between j-th animal and its i-th ancestor.

The higher the value of the average equivalent of complete generations (EqG), the better the quality of the pedigree information.

We estimated the individual inbreeding coefficients (F_X_) for all examined animals according to Meuwissen and Luo [7]. The genetic contributions of founders and ancestors to the two reference populations of cows—20,827 Simmental cows born between 1984 and 2009 (subset A) and 19,554 cows born between 2010 and 2020 (subset B)—were estimated according to the formulas of Lacy [31] and Boichard et al. [30], as described in Kania-Gierdziewicz et al. [21]. The SAS statistical package (proc FREQ) (version SAS 9.4) was used to examine the relationship between the number of inbred cows in particular birth periods. The significance of differences between subsets A and B in the values of ancestral inbreeding coefficients was examined using the non-parametric U Mann–Whitney test for independent groups (NPAR1WAY procedure in the SAS package) [32].

For estimation of the ancestral inbreeding coefficients, we identified the main founders and ancestors with the highest genetic contribution to both subsets. The ancestral inbreeding coefficients for cows from both reference populations with respect to the main founders and ancestors were calculated using the grain procedure from Boichard’s PEDIG V6 package [33]. The inbreeding coefficient (F_C_) is the proportion of loci that are “identical by descent” in an animal’s genome. The grain procedure calculates F_C_ as the total number of replications assigned as “identical by descent” divided by the total number of replications in the gene dropping process. Ancestral inbreeding coefficients were estimated using algorithms proposed by Ballou [18] and Kalinowski et al. [19], which are implemented in the grain program. Ancestral inbreeding according to Ballou [18] is calculated by the following formula:

$$F_{Bal(X)}=\frac{\left[F_{Bal(S)}+\left(1-F_{Bal\left(S\right)}\right)\cdot F_{Wright(S)}+F_{Bal(D)}+\left(1-F_{Bal\left(D\right)}\right)\cdot F_{Wright(D)}\right]}{2}$$

where S and D are the sire and dam of individual X, and F_Wright_ is the inbreeding coefficient (F_Wright(S)_ individual inbreeding coefficient for sire and F_Wright(D)_ for dam) obtained according to Wright’s formula [16]:

$$F_{Wright}=\sum_{i=1}^{n}(1+F_{i}){\left(\frac{1}{2}\right)}^{k_{S}+k_{D}+1}$$

where

n—number of paths the sire of animal X with the dam of X through the i-th common ancestor,

F_i_—inbreeding coefficient of common ancestor i,

k_S_—number of generations from sire (included) to common ancestor i (excluded),

k_D_—number of generations from dam (included) to common ancestor i (excluded).

Ancestral inbreeding according to Ballou (F_Bal_) is the probability that any allele in a given animal has been autozygous—that is, “identical by descent”—at least once in previous generations due to inbreeding. F_Bal_ is the proportion of alleles already marked out of all alleles in the given animal’s genome. The grain procedure calculates F_Bal_ as the total number of marked alleles divided by the total number of genome alleles (two times the total number of replications in the gene dropping process).

The ancestral inbreeding coefficient (F_Kal_) according to Kalinowski et al. [19] represents the historical inbreeding value, where two alleles are in a homozygous state if they have met previously in the population’s history. It can be defined as the probability that any allele was autozygous—that is, “identical by descent”—at least once in previous generations and is currently autozygous in a given individual. This ancestral inbreeding coefficient occurs only when common ancestors appear on both sides of the pedigree. F_Kal_ is the proportion of currently “identical by descent” alleles in an individual’s genome, already marked if these occurred as “identical by descent” in the past. The grain procedure calculates F_Kal_ as the total number of marked alleles currently “identical by descent” divided by two times the total number of replications in the gene dropping process:

$$F_{Kal(X)}=\frac{n_{marked,IBD}}{2R}$$

where

n_marked,IBD_—total number of replications in which the sampled allele is currently “identical by descent” (IBD) and was also IBD in at least one ancestor of animal X,

R—total number of replications in the gene dropping process.

Kalinowski’s new inbreeding coefficient (F_New_) is obtained by subtracting F_Kal_ from F_C_, because the ancestral and new inbreeding components sum to total inbreeding:

$$F_{New(X)}=F_{C}-F_{Kal(X)}$$

The ancestral history coefficient (AHC) according to Baumung et al. [20] takes into account how frequent an allele randomly taken from a given animal’s genome was in “identical by descent” status during pedigree segregation through gene dropping. This is performed by flagging alleles the first time they are “identical by descent” and keeping this information through the replications. AHC is a coefficient of ancestral inbreeding that measures the probability of purging deleterious recessive alleles, not through selection but through the increase in the number of “identical by descent” alleles by gene dropping during pedigree segregation. AHC is the sum of all “identical by descent” occurrences for each allele of an ancestor of a given animal in the gene dropping process during pedigree segregation. This sum is then divided by the total number of genome alleles (two times the total number of replications in the gene dropping process):

$${AHC}_{\left(x\right)}=\frac{\sum_{r=1}^{R}\sum_{a=1}^{2}c_{r,a}}{2R}$$

where

R—total number of replications in the gene dropping process,

a—index of the two alleles (1 and 2) of individual X,

c_r,a_—number of times allele a has been in the “identical by descent” state up to and including replication r.

By definition, the F_C_, F_Bal_, F_Kal_, and F_New_ coefficients must take values between 0 and 1, but the AHC value can exceed 1. The F_C_, F_Bal_, F_Kal_, and AHC coefficients were estimated after 100,000 iterations.

## 3. Results and Discussion

### 3.1. Quality of Pedigrees and the Inbreeding Coefficient Values

In the analyzed Simmental cow population, pedigree completeness was satisfactory. The EqG values for all cows ranged from 3.53 in subset A to 4.67 in subset B. Corresponding EqG values for inbred cows were 4.42 and 5.03 for cows from subsets A and B, respectively. The level of ancestor identification up to the fourth generation in the pedigrees of Simmental cows was satisfactory across all generations and sufficient for F_X_ calculation; however, slightly less information was recorded on the dam side of the pedigree [21].

**Figure 1:**
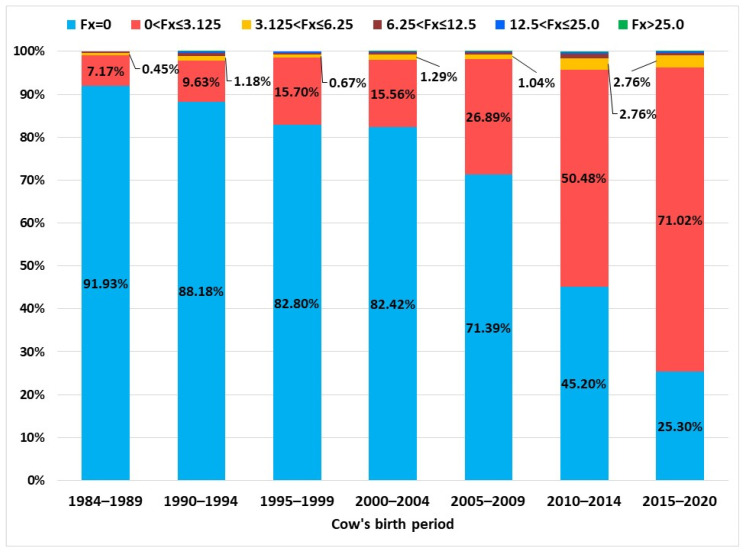
Percentage of cows with various inbreeding coefficients in consecutive birth periods.

The mean inbreeding coefficients were generally low in both reference populations: in subset A they were 0.32% for all cows and 1.36% for inbred cows; and in subset B they were 0.68% and 1.06%, respectively. However, about three times more cows were inbred in subset B than in subset A. Over 70% of all inbred cows were born in the last 10 years. On the other hand, only slightly over 30% of all non-inbred cows were born in the last ten years. Moreover, over 60% of cows born between 2010 and 2020 had an inbreeding coefficient higher than 0, including three cows with an inbreeding level exceeding 31.25% (Figure 1). The relationship between the number of inbred cows in consecutive birth periods was highly significant (χ^2^ = 8134, *p* < 0.0001).

Similar results were reported by Török et al. [10] for the Hungarian population of Simmental cows. Among the 11,476 Hungarian Simmental cows born after 1997, 7165 were inbred, and average inbreeding was 1.1%. The low overall inbreeding level was largely due to the fact that only 48 individuals showed inbreeding higher than 10%. Both in Török’s study and in subset B of the present study, the percentage of inbred animals was approximately 63%. A somewhat similar pattern of cow distribution across inbreeding coefficient classes, particularly for inbreeding coefficients higher than 20%, was reported by de Fatima Sieklicki et al. [2] for Holstein cattle in Brazil and by Jarnecka et al. [34] for the Polish Red cattle population. Strapakova and Strapak [35] described a similar distribution across inbreeding classes for Slovak Simmental cattle. They found that the percentage of animals with an inbreeding coefficient equal to zero (F = 0) decreased from 82.9% in 1995–1999 to 1.82% in 2015–2021, while the percentage of cows with inbreeding values up to 3.125% increased over the same period from 16.25% to 86.99%.

### 3.2. Founders’ and Ancestors’ Gene Contribution and Inbreeding Subdivision

Table 1 shows the distribution of founders by gene contribution and sex, and Table 2 presents the distribution of ancestors. In the pedigrees of the older cows (subset A), there were a total of 14,755 founders and 11,103 ancestors. In the pedigrees of cows born between 2010 and 2020 (subset B), there were a total of 23,780 founders and 18,965 ancestors. The maximum genetic contribution of the main founders reached 1.5% and 2.3% in cows from subsets A and B, respectively. For the main ancestors, the corresponding values were 2.7% and 4.7% in cows from subsets A and B, respectively.

For Mexican Simmental cattle, Rios Utrera et al. [7] found a similar maximum genetic contribution for the main ancestors but a slightly higher number of main ancestors (15 individuals). Worede et al. [36] also found a similar maximum genetic contribution for the Brown Swiss cattle population, but with more main ancestors (20 individuals). Melka et al. [37] reported a higher genetic contribution of the first main ancestor for Guernsey cattle, ranging from 13.9% to 17.1% depending on the population. Examining Canadian dairy cattle breeds in another study, Melka et al. [11] found much higher maximum values for ancestor genetic contribution, ranging from 10.7% for the Brown Swiss breed to 21.5% for Ayrshire cattle. For German Brown cattle, Wirth et al. [38] obtained a higher maximum genetic contribution of the first main ancestor, approximately 9%. For Mexican Romosinuano cattle, Núñez-Domínguez et al. [3] reported a much higher genetic contribution of the first main founder, exceeding 30%.

Table 3 shows the contribution of the main founders and ancestors to inbreeding in both reference populations. As shown there, seven of the same founders (3 sires and 4 cows) appeared in both subsets. In subset A there were also three founders that did not occur in subset B. These main founders were born between 1972 and 1984. Almost all were of foreign origin, imported in most cases from Austria or Germany. Only two founders in subset A were of Polish origin. Seven of the founders were purebred Simmentals, and the remainder were crossbred animals with a predominant contribution of Simmental genes.

In Table 4 the same seven founders appear in the pedigrees of cows in both subsets (A and B), but they occur more frequently in the pedigrees of cows born between 2010 and 2020 (subset B), as evidenced by the higher average inbreeding values in this group. The last three founders, two sires and one dam appearing only in subset A, had only marginal significance for the inbreeding subdivision, as evidenced by their absence in subset B. The percentage of occurrences of each founder common to both subsets in the pedigrees of young cows (subset B) ranged from approximately 42% to 45% for F_C_, depending on the founder. The corresponding percentages ranged from 42% to 49% for F_Bal_, from 44% to 49% for F_Kal_, and from 42% to 49% for AHC, depending on the founder. The average F_New_ values for cows from subset B were higher than for cows from subset A, indicating that some problems with inbreeding had already occurred in the main founders.

Table 3 also shows the main ancestors for both reference populations of cows. The main ancestors were born between 1977 and 2006. In total, 20 animals were identified as main ancestors across both subsets of cows. Of these, 18 were sires and 2 were dams. For cows from subset A, there were 11 main ancestors (all males). All were purebred Simmental sires; 9 of them were of Polish origin and 2 were imported from Germany. In the reference population of young cows from subset B there were 15 main ancestors (13 sires and 2 cows). Of these, 12 were purebred Simmentals and 3 were crossbred animals with a high proportion of Simmental genes. All but three of those ancestors were of Polish origin. These three foreign ancestors were imported from Germany. Six ancestors were common to both reference populations, nine occurred only in the pedigrees of young cows (subset B), and five occurred only in subset A (older cows).

The same pattern as for main founders can be observed in Table 5 for the six ancestors common to both reference populations of cows (subsets A and B). They appeared more frequently in the pedigrees of young cows (subset B), resulting in higher average inbreeding values in this group. In addition, the youngest ancestor, the sire born in 2006, appeared on both sides of the pedigrees of only nine animals, likely extending no further back than the second generation. Hence, apart from F_C_, all other partial inbreeding coefficients for this sire are equal to zero. The second sire, born in 1996, did not appear deeper than 4–5 generations back in the pedigrees, so its F_Kal_ value is equal to zero. Such high F_c_ values for three ancestors (Table 5) were probably because they appeared several times in the pedigrees of a small number of young animals as sires and grandsires. The percentage of occurrences of each ancestor common to both subsets in the pedigrees of young cows (subset B) could be estimated only for the aforementioned six common ancestors. This percentage ranged from 31% to 47% for F_C_ and from 35% to 68% for F_Bal_, depending on the ancestor. The corresponding percentages for F_Kal_ ranged from 14% to 56% and for AHC from 35% to 68%, depending on the ancestor. The average F_New_ values for cows from subset B were almost six times higher than for cows from subset A, indicating that the main ancestors were responsible for the inbreeding increase in young cows born between 2010 and 2020 (subset B).

More complete pedigrees in younger cows (subset B) could also have contributed to higher estimates of founders’ and ancestors’ contributions to inbreeding Fc, F_Bal_, F_Kal_, F_New_, and AHC in Table 3, Table 4 and Table 5.

For German Brown cattle, Wirth et al. [38] found an inbreeding coefficient of 0.023, the Ballou inbreeding coefficient of 0.022, the Kalinowski inbreeding coefficient of 0.002, and the Baumung AHC coefficient of 0.023. The inbreeding coefficients they reported are much lower than the totals we obtained here for the main founders and ancestors of Polish Simmental cows. This indicates that the main founders and ancestors of Simmental cows appeared much more frequently on both sides of cows’ pedigrees and across many pedigrees. However, the inbreeding subdivision in our present study is quite different from that found for German Brown cattle by Wirth et al. [38]. For 35 German sheep breeds, Justinski et al. [39] found a mean Kalinowski inbreeding coefficient of 0.022 and a mean Ballou inbreeding coefficient of 0.082; they also reported a mean Baumung AHC coefficient of 0.090. They concluded that their analysis across 35 breeds revealed losses of genetic diversity, mainly due to unequal contributions of founders in the past, and that this influenced the level of the new inbreeding coefficient more than the levels of ancestral or classical inbreeding coefficients. Their values of the Kalinowski and Ballou inbreeding coefficients and the Baumung AHC coefficients were similar to or lower than ours, but they were calculated in a different way—for entire sheep breeds and for all ancestors within each breed.

## 4. Conclusions

Summarizing, the ten main founders identified in subset A and the seven main founders in subset B, with a maximum individual genetic contribution of 2.3%, together with the 20 main ancestors, whose maximum genetic contribution reached 4.7% in young cows, collectively drove the observed increase in inbreeding. The six sires (ancestors) common to both reference populations appeared with increasing frequency in the pedigrees of young cows (subset B), as reflected in the higher values of all partial inbreeding coefficients (F_C_, F_Bal_, F_Kal_, and AHC) and especially F_New_ in that group. The rising prevalence of inbred cows, with over 70% of all inbred animals born in the last ten years, confirms that the current breeding strategy has not been effective in controlling the accumulation of inbreeding.

The increase in the number of ancestors resulting from the use of imported animals did not lead to greater genetic diversity; this indicates that the imported animals were often related to those already present in the population. Such a finding highlights the importance of not only increasing the number of imported animals but also ensuring that they originate from genetically distinct lineages. Pedigree analysis, combined with calculation of ancestral inbreeding coefficients, proved to be a useful tool for identifying the sources of inbreeding accumulation and should be applied routinely in breeding programs for the Polish Simmental cattle population. To prevent further increases in inbreeding and to maintain long-term genetic diversity in the population, it is recommended to monitor the pedigrees of both domestically bred and imported animals, with particular attention to the representation of key founders and ancestors on both sides of the pedigree.

## Figures and Tables

**Table 1:** Distribution of founders by genetic contribution and sex in subsets A and B of cows.

Founders	Contribution of Genes [%]			
	<0.1	(0.1, 0.5]	(0.5, 1.0]	>1.0
Subset A ^(1)^				
Male	1731	65	9	2
Female	12,844	94	10	0
Total	14,575	159	19	2
Subset B ^(2)^				
Male	2125	33	8	3
Female	21,505	84	18	4
Total	23,630	117	26	7

^(1)^ Cows born between 1984 and 2009; ^(2)^ Cows born between 2010 and 2020.

**Table 2:** Distribution of ancestors by sex and genetic contribution in subsets A and B of cows.

Ancestors	Contribution of Genes [%]			
	<0.1	(0.1, 0.5]	(0.5, 1.0]	>1.0
Subset A ^(1)^				
Male	435	73	13	11
Female	10,531	36	4	0
Total	10,966	109	17	11
Subset B ^(2)^				
Male	854	72	12	13
Female	17,959	48	5	2
Total	18,813	120	17	15

^(1)^ Cows born between 1984 and 2009; ^(2)^ Cows born between 2010 and 2020.

**Table 3:** Average contribution of main founders and ancestors to inbreeding of animals from both reference populations (subsets A and B).

	Subset A ^(1)^	Subset B ^(2)^	Mann–Whitney U Statistic	*p*-Value ^(3)^
Number of main founders	10	7		
Number of main founders common to both subsets	7	7		
Contribution of major founders to the following:				
Classical inbreeding coefficient (F_C_)	0.003	0.005	429.3	<0.0001 ^(4)^
Ballou’s ancestral inbreeding coefficient (F_Bal_)	0.003	0.006	418.3	<0.0001
Kalinowski’s ancestral inbreeding coefficient (F_Kal_)	0.0001	0.0002	406.2	<0.0001
Kalinowski’s new inbreeding coefficient (F_New_)	0.003	0.005	419.2	<0.0001
Baumung’s ancestral history coefficient (AHC)	0.003	0.006	418.3	<0.0001
Number of main ancestors	11	15		
Number of main ancestors common to both subsets	6	6		
Contribution of major ancestors to the following:				
Classical inbreeding coefficient (F_C_)	0.003	0.004	416.7	<0.0001
Ballou’s ancestral inbreeding coefficient (F_Bal_)	0.003	0.005	413.7	<0.0001
Kalinowski’s ancestral inbreeding coefficient (F_Kal_)	0.001	0.0002	402.5	<0.0001
Kalinowski’s new inbreeding coefficient (F_New_)	0.002	0.004	415.7	<0.0001
Baumung’s ancestral history coefficient (AHC)	0.003	0.005	413.7	<0.0001

^(1)^ Cows born between 1984 and 2009; ^(2)^ Cows born between 2010 and 2020; ^(3)^ *p*-value—probability (significance) of the test; ^(4)^ differences significant if *p*-value < 0.05, differences highly significant if *p*-value < 0.01.

**Table 4:** Contribution of main founders to inbreeding of animals from both reference populations (subsets A and B).

Founder’s ID (Sex)	Founder’s Origin	Subset A ^(1)^				Subset B ^(2)^			
		F_C_	F_Bal_	F_Kal_	AHC	F_C_	F_Bal_	F_Kal_	AHC
896 (M)	Austria	0.002	0.002	0.0003	0.002	0.006	0.007	0.001	0.007
208 (M)	Austria	0.004	0.003	0.0005	0.003	0.012	0.010	0.002	0.010
1607 (F)	Czech Rep.	0.004	0.003	0.0005	0.003	0.012	0.010	0.002	0.011
4352 (M)	Germany	0.010	0.008	0.001	0.008	0.034	0.022	0.003	0.022
3682 (F)	Germany	0.002	0.002	0.0003	0.002	0.008	0.007	0.001	0.007
157 (F)	Austria	0.004	0.002	0.0005	0.003	0.011	0.008	0.002	0.008
2878 (F)	Czech Rep.	0.002	0.002	0.0003	0.002	0.006	0.007	0.001	0.007
4938 (M)	Poland	0.008	0.004	0.0005	0.004				
1408 (F)	Czech Rep.	0.003	0.002	0.0006	0.002				
5585 (M)	Poland	0.025	0.007	0	0.007				

^(1)^ Cows born between 1984 and 2009; ^(2)^ Cows born between 2010 and 2020.

**Table 5:** Contribution of main ancestors to inbreeding of animals from both reference populations (subsets A and B).

Ancestor’s ID (Sex)	Ancestor’s Origin	Subset A ^(1)^				Subset B ^(2)^			
		F_C_	F_Bal_	F_Kal_	AHC	F_C_	F_Bal_	F_Kal_	AHC
4064 (M)	Germany	0.007	0.006	0.001	0.006	0.023	0.022	0.003	0.022
5717 (M)	Poland	0.003	0.003	0.0004	0.004				
7406 (M)	Poland					0.022	0.025	0.003	0.025
8208 (M)	Poland	0.007	0.005	0.001	0.005	0.023	0.015	0.003	0.015
3957 (M)	Germany	0.004	0.004	0.001	0.004	0.011	0.015	0.002	0.015
7578 (M)	Poland	0.031	0.025	0.004	0.026	0.108	0.071	0.010	0.071
8769 (M)	Poland	0.044	0.023	0.003	0.023	0.152	0.090	0.031	0.090
7954 (M)	Poland	0.006	0.005	0.001	0.005	0.019	0.013	0.001	0.013
5079 (M)	Poland	0.015	0.009	0.001	0.009				
7350 (M)	Poland	0.004	0.004	0.001	0.004				
5423 (M)	Poland	0.001	0.002	0.0004	0.002				
5695 (M)	Poland	0.017	0.011	0.003	0.011				
6401 (M)	Poland					0.012	0.011	0.001	0.011
6045 (M)	Poland					0.016	0.013	0.003	0.013
12867 (M)	Poland					0.194	0.000	0.000	0.000
11615 (M)	Poland					0.069	0.062	0.000	0.062
10005 (F)	Poland					0.030	0.021	0.010	0.021
9543 (M)	Poland					0.074	0.047	0.006	0.047
4246 (M)	Germany					0.004	0.003	0.001	0.003
9645 (F)	Poland					0.041	0.021	0.007	0.021

^(1)^ Cows born between 1984 and 2009; ^(2)^ Cows born between 2010 and 2020.

## Data Availability

The data were not deposited in an official repository. Further information regarding the data and models will be made available by the author upon request for correspondence.
